# Supplementary material for: From Primary MSC Culture of Adipose Tissue to Immortalized Cell Line Producing Cytokines for Potential Use in Regenerative Medicine Therapy or Immunotherapy
Source: Int J Mol Sci. 2021 Oct 23;22(21):11439. doi: 10.3390/ijms222111439 (PMC8584013; doi:10.3390/ijms222111439)
Supplement: Supplementary file 1 [file ijms-22-11439-s001.zip › ijms-1422471-supplementary.pdf]

Suplementary data

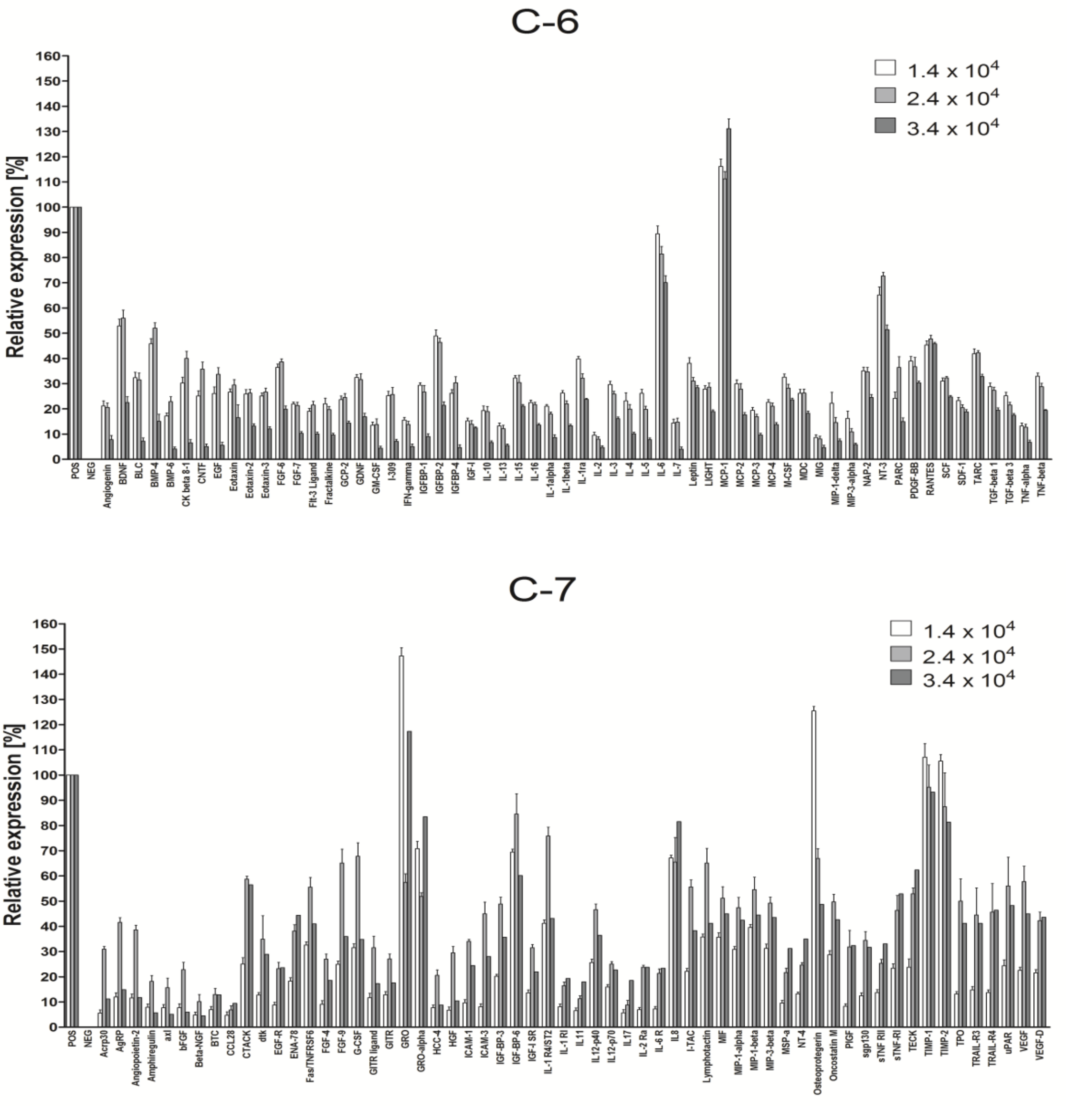

Figure S1. Cytokine production as a function of the initial cell culture density. Secretion of cytokines was evaluated using RayBio® C Human Cytokine Antibody Array for a panel of 120 cytokines and expressed as histogram relative to positive control. Cell cultures were established starting from densities: 1.4 x 10^4, 2.4 x 10^4 and 3.3 x 10^4 cells plated per cm^2 area of the culture vessel. The data represent the mean from a duplicate assessement ±SEM.

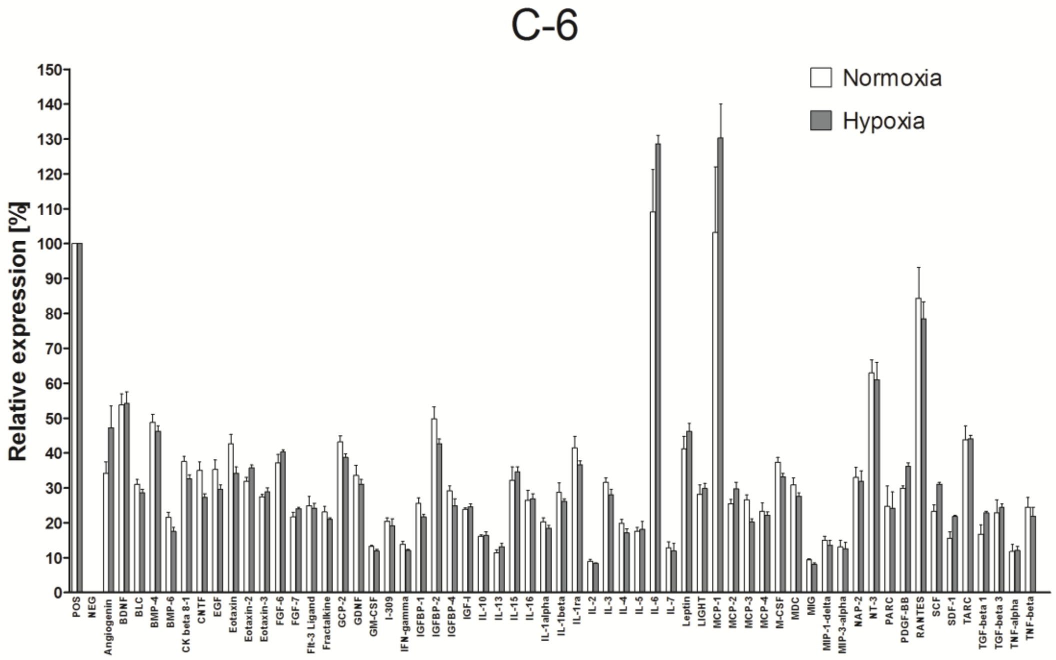

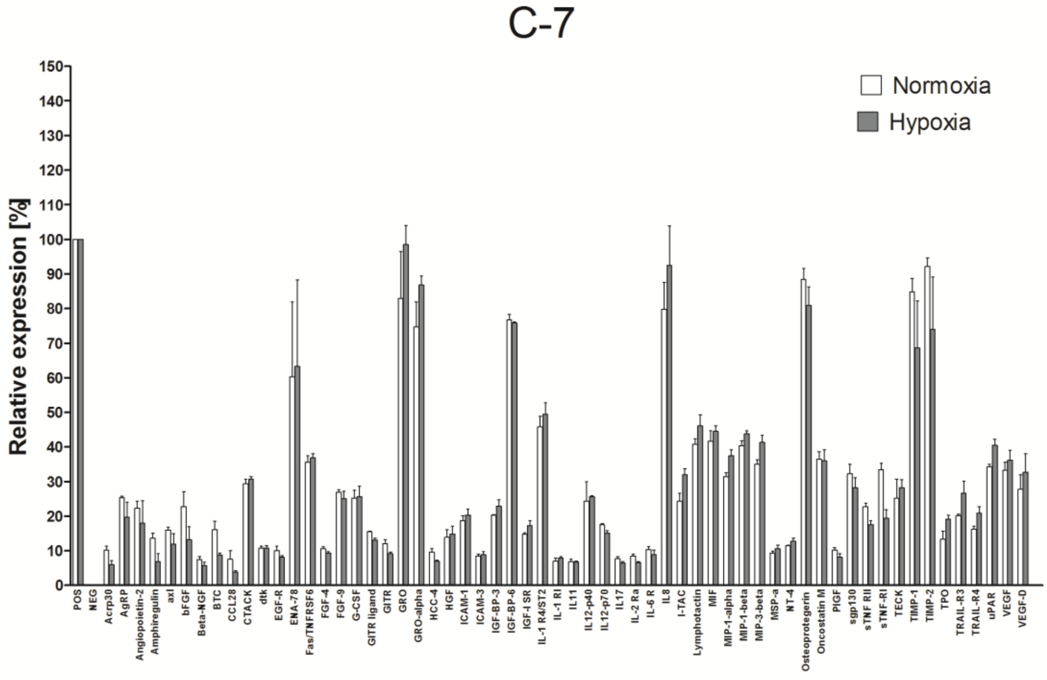

Figure S2. Cytokine production as a function of culture under normoxic and hypoxic conditions. The secretory profile of the tested line was determined using the RayBio C-series Human Cytokine Antibody Array Array for a panel of 120 cytokines and expressed as histogram relative to positive control. The data represent the mean from a duplicate assessement ±SEM.

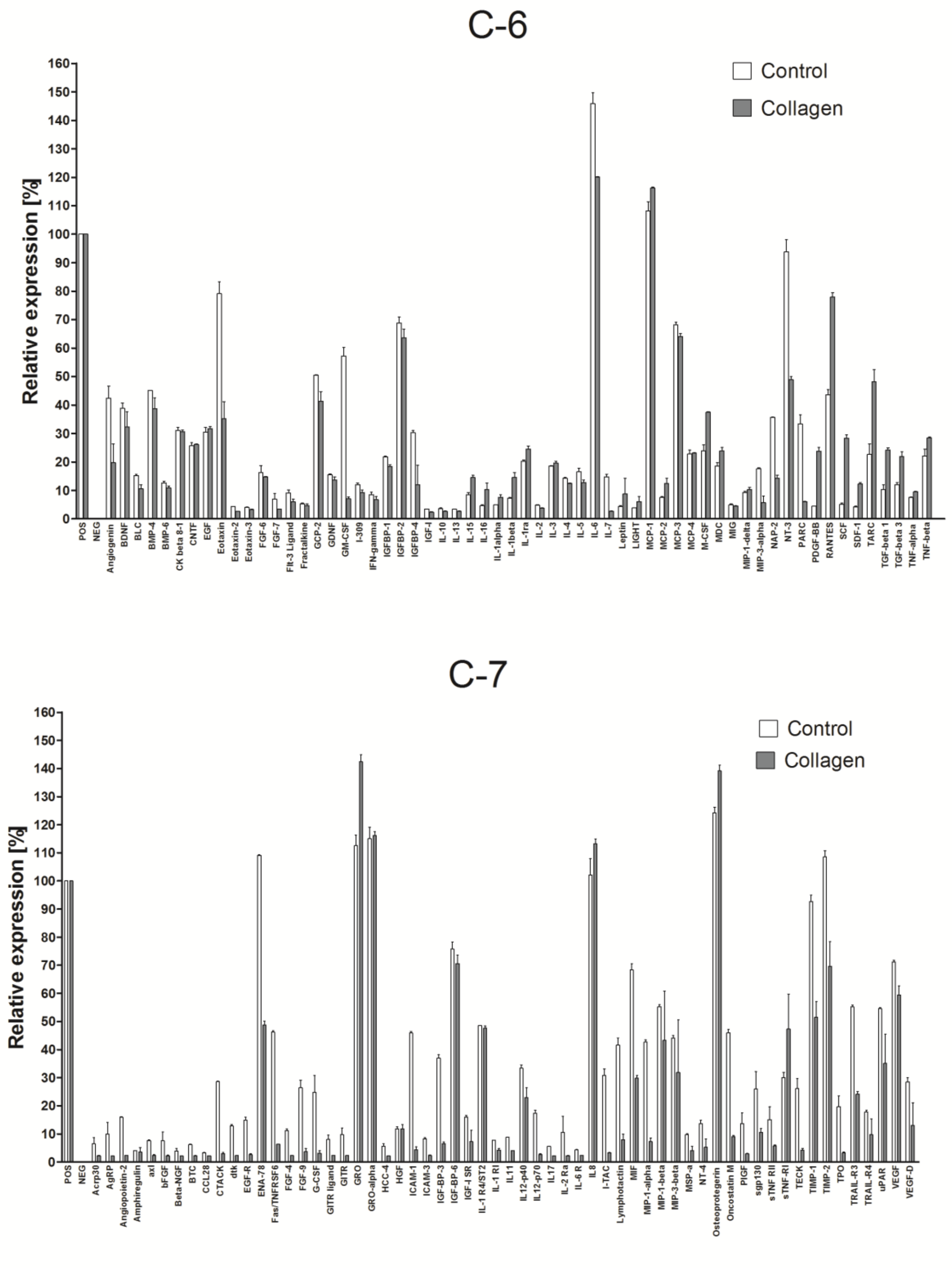

Figure S3. Cytokine production as a function of culture on collagen disc and extended culture time. The secretory profile of the tested line was determined using the RayBio C-series Human Cytokine Antibody Array Array for a panel of 120 cytokines and expressed as histogram relative to positive control. The data represent the mean from a duplicate assessement and mean ±SEM.

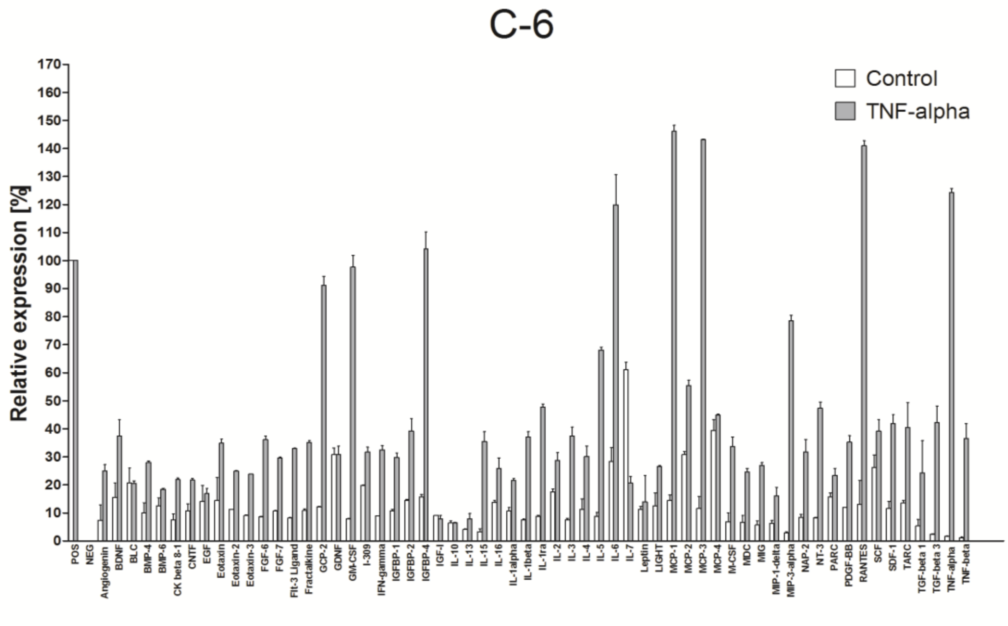

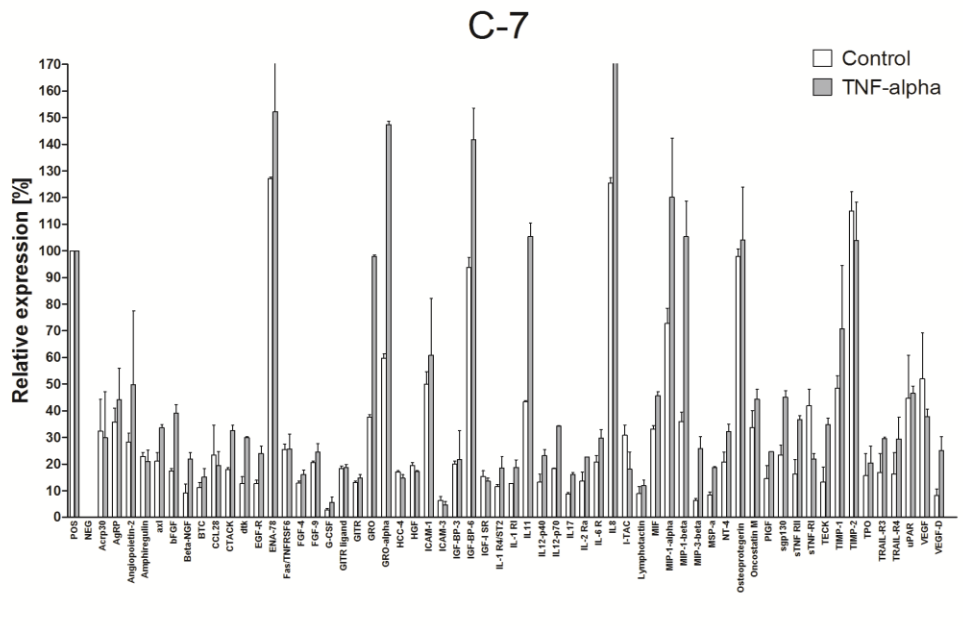

Figure S4. Cytokine production after 24 h TNF-alpha stimulation of cell culture. The secretory profile of the tested line was determined using the RayBio C-series Human Cytokine Antibody Array Array for a panel of 120 cytokines and expressed as histogram relative to positive control. Cell cultures were established and stimulated with 50 µg/mL of TNF-alpha to receive supernatants. The data represent the mean from a duplicate assessement ±SEM.

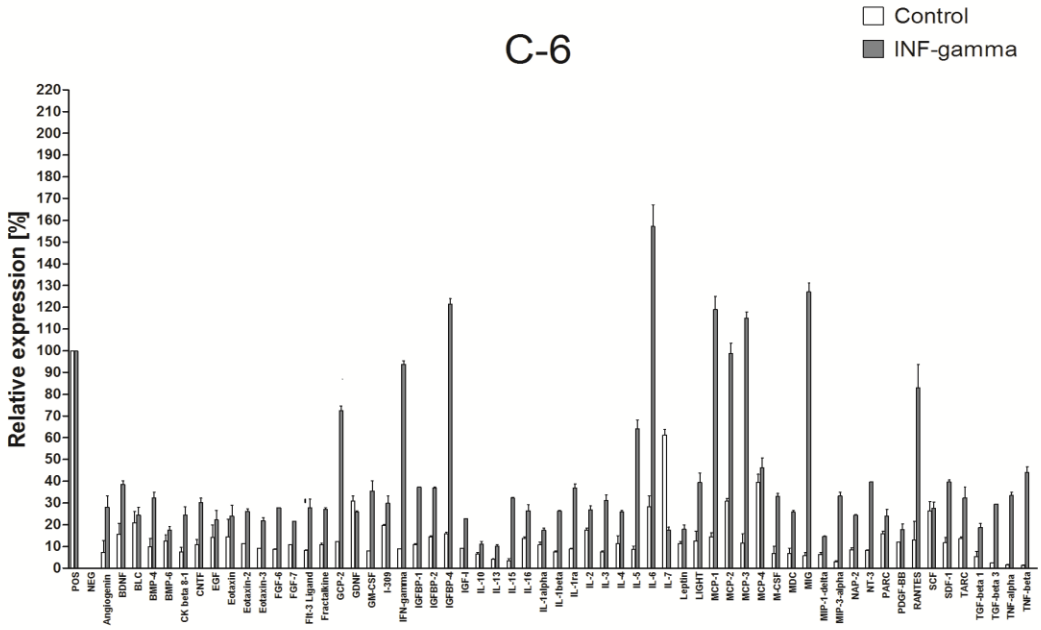

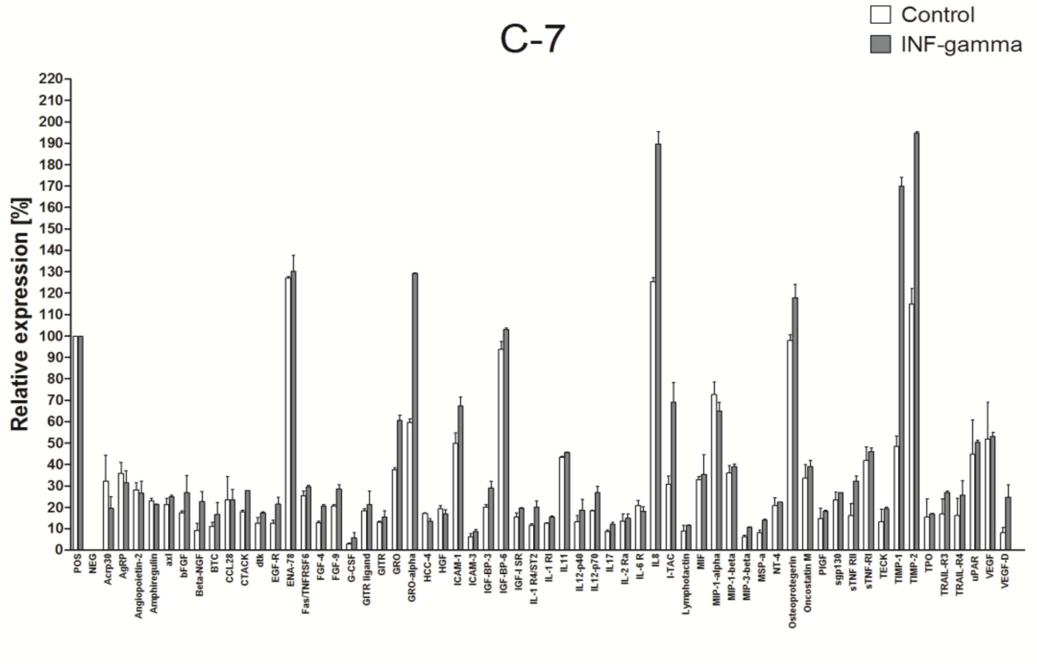

Figure S5. Cytokine production after 24 h INF-gamma stimulation of cell culture. The secretory profile of the tested line was determined using the RayBio C-series Human Cytokine Antibody Array Array for 120 cytokines and expressed as histogram relative to positive control. Cell cultures were established and stimulated with 50 μg/mL of INF-gamma to receive supernatants. The data represent the mean from a duplicate assessement ±SEM.

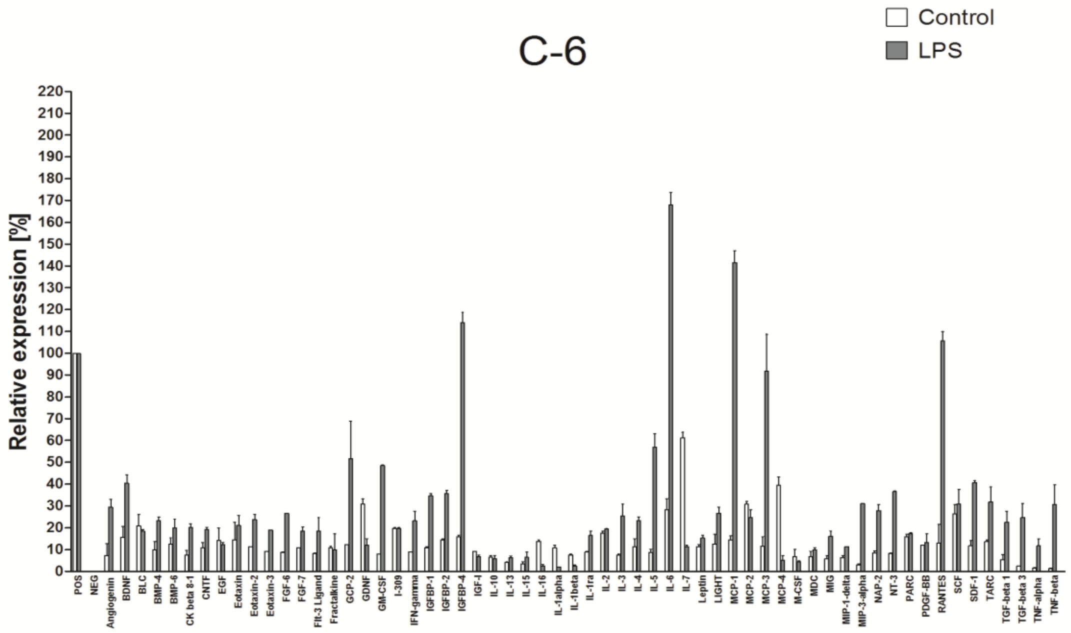

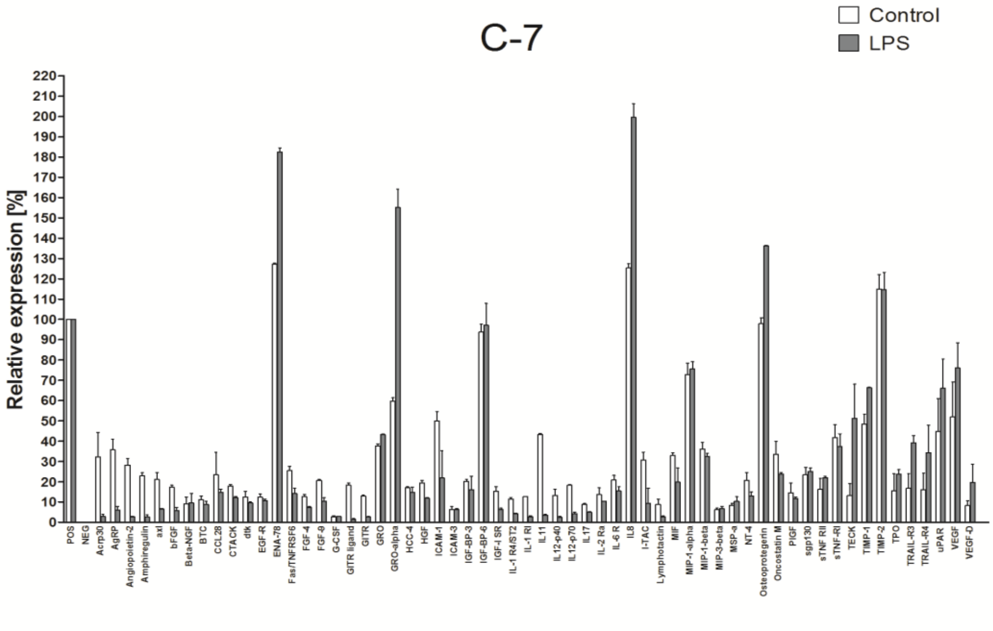

Figure S6. Selected cytokine production after 24 h LPS stimulation of cell culture. The secretory profile of the tested line was determined using the RayBio C-series Human Cytokine Antibody Array Array for a panel of 120 cytokines and expressed as histogram relative to positive control. Cell cultures were established and stimulated with 100 μg/mL of LPS to receive supernatants. The data represent the mean from a duplicate assessement ±SEM.

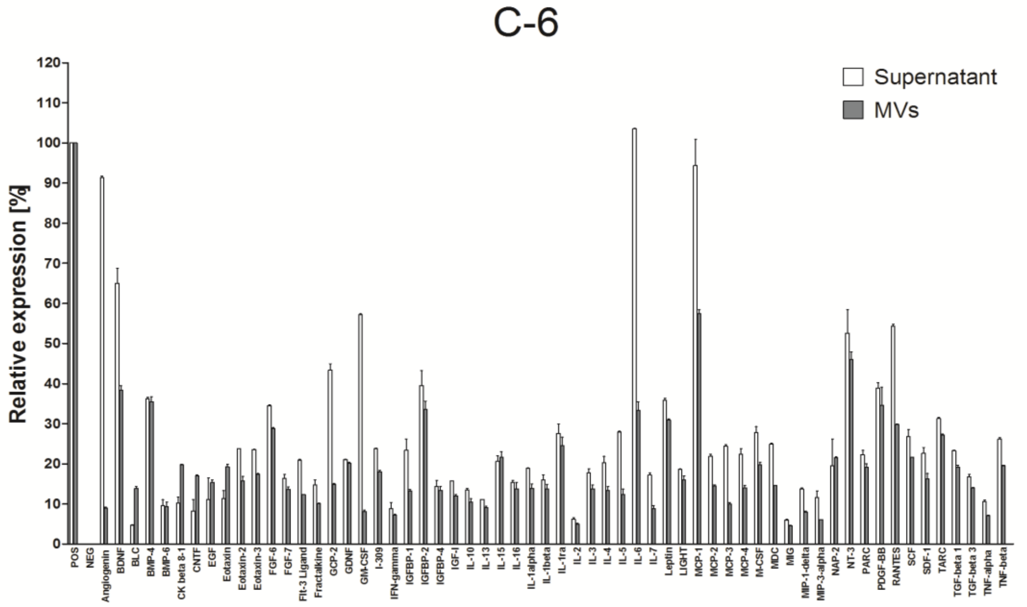

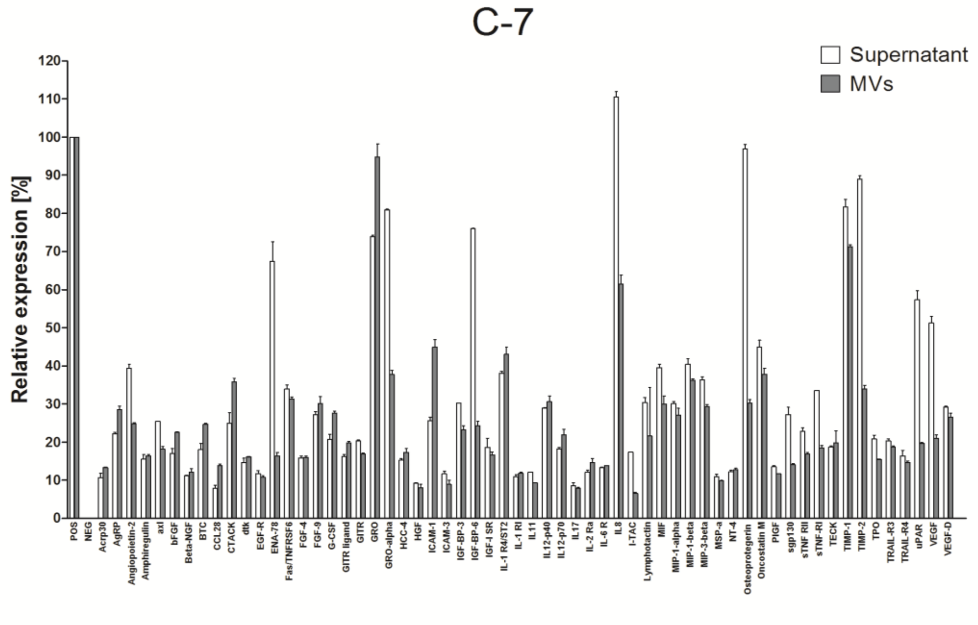

Figure S7. Cytokine production in the free form (Supernatant) and in the microvesicles (MVs) form. The secretory profile of the tested line was determined using the RayBio C-series Human Cytokine Antibody Array Array for a panel of 120 cytokines and expressed as histogram relative to positive control. The MVs present in the supernatant were obtained by sequential centrifugations. They were lysed 100%to evaluate cytokine concentration. The data represent the mean from a duplicate assessement ±SEM.
